# On the group theoretical background of assigning stepwise mutations onto phylogenies

**DOI:** 10.1186/1748-7188-7-36

**Published:** 2012-12-15

**Authors:** Mareike Fischer, Steffen Klaere, Minh Anh Thi Nguyen, Arndt von Haeseler

**Affiliations:** 1 Department for Mathematics und Computer Science, Ernst-Moritz-Arndt-University Greifswald, Walther-Rathenau-Strasse 47, 17487 Greifswald, Germany; 2 Department of Statistics and School of Biological Sciences, University of Auckland, Private Bag 92019, Auckland, New Zealand; 3 Groningen Bioinformatics Centre, University of Groningen, Nijenborgh 7, 9747 AG Groningen, The Netherlands; 4, Center for Integrative Bioinformatics ViennaMax F. Perutz Laboratories, University of Vienna, Medical University of Vienna, University of Veterinary Medicine Vienna, Dr. Bohr Gasse 9, A-1030, Vienna, Austria

**Keywords:** Maximum likelihood, Maximum parsimony, Substitution model, Tree reconstruction, Group theory

## Abstract

Recently one step mutation matrices were introduced to model the impact of substitutions on arbitrary branches of a phylogenetic tree on an alignment site. This concept works nicely for the four-state nucleotide alphabet and provides an efficient procedure conjectured to compute the minimal number of substitutions needed to transform one alignment site into another. The present paper delivers a proof of the validity of this algorithm. Moreover, we provide several mathematical insights into the generalization of the OSM matrix to multi-state alphabets. The construction of the OSM matrix is only possible if the matrices representing the substitution types acting on the character states and the identity matrix form a commutative group with respect to matrix multiplication. We illustrate this approach by looking at Abelian groups over twenty states and critically discuss their biological usefulness when investigating amino acids.

## Background

Alignments of homologous sequences provide fundamental materials to the reconstruction of phylogenetic trees and many other sequence-based analyses (see, e.g., [1,2]). Each alignment column (site) consists of character states that are assumed to have evolved from a common ancestral state by means of substitutions. Any combination of the character states in the aligned sequences at one alignment column represents a so-called *character*[3], which is sometimes also called *site pattern*[4]. Given a phylogenetic tree and an alignment that evolved along the tree, Klaere et al. [5] showed, for binary alphabets, how a character changes into another character if a substitution occurs on an arbitrary branch of the tree. The impact of such a substitution is summarized by the so-called *One Step Mutation* (OSM) matrix. The OSM matrix allows for analytical formulae to compute the posterior probability distribution of the number of substitutions on a given tree that give rise to a character [5].

Nguyen et al. [4] extended the concept of the OSM matrix to the four-state nucleotide alphabet while developing a method, the Misfits algorithm, to evaluate the goodness of fit between models and data in phylogenetic inference. There, the OSM matrix is constructed based on the Kimura three parameter (K3ST) substitution model [6]. Nguyen et al. [4] illustrated how one can apply the Fitch algorithm [7] to compute the minimal number of substitutions required to change one character into another character under the OSM setting. In the present paper, we deliver a proof of the validity of this algorithm.

In addition, the OSM matrix can be constructed only if the matrices representing the substitutions, the so-called *substitution matrices,* and the identity matrix form a commutative or Abelian group (see, e.g., [8]) with respect to matrix multiplication [4]. The link between Abelian groups in phylogenetic models has been studied before, most notably by Hendy et al. [9]. Further, an extension of nucleotide substitution models with an underlying Abelian group to joint states at the leaves of a tree has also been studied by other authors. Bashford et al. [10] introduced an approach very similar to OSM to study the multi-taxon tensor space. Bryant [11] also introduced a very similar framework to study the Hadamard transform of [12] in the light of multi-taxon processes.

In this work, we first introduce standard phylogenetic notation. We then formalize the construction of the OSM matrix, and which part of its construction is used in the Misfits algorithm. We further present possible extensions of the OSM framework to arbitrary alphabets. We will show that the Misfits algorithm in fact computes the minimal number of substitutions needed to change one character into another character. Moreover, we discuss the extension of the algorithm to substitution models which do not have an underlying Abelian group. Finally, we discuss the Abelian groups available for amino acids.

## Notation and problem recapitulation

### Notation

Recall that a *rooted binary phylogenetic X-tree* is a tree $\left(T=(V(T),E(T))\right)$ with the following properties: There is one vertex $\left(\rho \in V(T)\right)$ with indegree 0 and outdegree 1, which is called the *root* of $\left(T\right)$. All edges $\left(e\in E(T)\right)$ are directed away from *ρ*, and all vertices $\left(v\in V(T)\setminus \{\rho \}\right)$ have indegree 1 and outdegree 0 or 2. Vertices with outdegree 0 are usually referred to as *leaves* of $\left(T\right)$. Remember that for an *X*-tree, there are exactly |*X*|=*n*leaves, which is why there is a bijection between the set of leaves of $\left(T\right)$ and the taxon set *X*. Thus, when there is no ambiguity, we use the terms *leaf * and *taxon* synonymously. Moreover, we often just write “phylogenetic tree” or “tree” when referring to a rooted binary phylogenetic tree.

Furthermore, recall that a *characterf*is a function $\left(f:X\to C\right)$ for some set $\left(C:=\{c_{1},c_{2},c_{3},\ldots ,c_{r}\}\right)$ of *rcharacter states* ($\left(r\in N\right)$). We denote by $\left(C^{n}\right)$ the set of all *r*^*n*^ possible characters on $\left(C\right)$ and *n* taxa. For instance, for the four-state DNA alphabet, $\left(C_{\mathrm{DNA}}=\{A,G,C,T\}\right)$ and the set $\left(C_{\mathrm{DNA}}^{n}\right)$ consists of all 4^*n*^possible characters.

An *extension* of *f*to $\left(V(T)\right)$ is a map $\left(g:V(T)\to C\right)$ such that *g*(*i*)=*f*(*i*) for all *i* in *X*. For such an extension *g*of *f*, we denote by $\left(l_{T}(g)\right)$ the number of edges *e*={*uv*} in $\left(T\right)$ on which a substitution occurs, i.e. where *g*(*u*)≠*g*(*v*). The *parsimony score* of *f*on $\left(T\right)$, denoted by $\left(l_{T}(f)\right)$, is obtained by minimizing $\left(l_{T}(g)\right)$ over all possible extensions *g*. Given a tree $\left(T\right)$ and a character *f*on the same taxon set, one can easily calculate the parsimony score of *f* on $\left(T\right)$ with the famous Fitch algorithm [7]. Moreover, when a character state changes along one edge of the tree, we refer to this state change as *substitution* or *mutation*. As for our purposes only so-called manifest mutations are relevant, i.e. those mutations that can be observed and are not reversed, we do not distinguish between mutations and substitutions, which is why we use these terms synonymously.

### Construction of the OSM matrix

We now introduce the OSM framework in a stepwise fashion. The aim of the OSM approach is to determine the effects a single mutation occurring on a rooted tree $\left(T\right)$ has on a character evolving on that tree.

The first task of this approach is to formalize the term mutation and its effects on a single character state in $\left(C\right)$. A mutation is an operation $\left(\sigma :\;C\to C\right)$ which is bijective, i.e. it satisfies the following condition: 

C1. For all $\left(c_{i}\in C\right)$ there is a $\left(c_{j}\in C\right)$ such that *σ*(*c*_*i*_)=*c*_*j*_, and if *σ*(*c*_*i*_)=*σ*(*c*_*j*_), then *c*_*i*_=*c*_*j*_.

This guarantees that a mutation affects a character state in a unique fashion. It is well-known that any bijective function on a finite discrete state set is a permutation (e.g., [13]). Thus, a mutation is a specific instance of a permutation applied to a character.

The next step is to select the set *Σ*of admissible permutations acting on $\left(C\right)$. It is mathematically convenient to select *Σ*such that it forms an Abelian group [9] with a regular (transitive and free) action on $\left(C\right)$. Hence, *Σ* satisfies the following conditions: 

C2. For every pair $\left(c_{i},c_{j}\in C\right)$ there is exactly one permutation *σ* ∈ *Σ* such that *σ* (*c*_*i*_) = *c*_*j*_, i.e., the action of *Σ*on $\left(C\right)$ is regular.

C3. For all *σ*_1_,*σ*_2_ ∈ *Σ* also the product *σ*_1_ ∘ *σ*_2_ ∈ *Σ*. Mathematically speaking, *Σ*is closed with respect to concatenation of its permutations.

C4. For all *σ*_1_,*σ*_2_∈*Σ*we have $\left(\sigma_{1}\circ \sigma_{2}=\sigma_{2}\circ \sigma_{1}\right)$ Thus, *Σ*is commutative, and hence the order in which we assign permutations is irrelevant for the outcome.

C5. There is an element *σ*_0_ ∈ *Σ* such that for all *σ*_1_ ∈ *Σ* we have $\left(\sigma_{1}\circ \sigma_{0}=\sigma_{0}\circ \sigma_{1}=\sigma_{1}\right)$, i.e. there exists a so-called neutral element, namely the identity, in *Σ*. For all $\left(c_{i}\in C\right)$ only *σ*_0_(*c*_*i*_) = *c*_*i*_, i.e. *σ*_*i*_is fixed point free for all *σ*_*i*_≠*σ*_0_.

C6. For every *σ*_1_ ∈ *Σ* there exists a *σ*_2_ ∈ *Σ* such that *σ*_1_ ∘ *σ*_2_ = *σ*_0_. Mathematically speaking, for every element of *Σ*there exists an inverse element. This guarantees that every permutation can be reversed within a single step.

C7. For all *σ*_1_,*σ*_2_,*σ*_3_ ∈ *Σ* we have *σ*_1_ ∘ (*σ*_2_ ∘ *σ*_3_) = (*σ*_1_ ∘ *σ*_2_) ∘ *σ*_3_ = *σ*_1_ ∘ *σ*_2_ ∘ *σ*_3_, i.e. the associative law holds.

It should be noted that any set of permutations is associative, i.e. satifies C7. Thus, for a set of permutations *Σ* to be Abelian with a regular action on $\left(C\right)$ it only needs to satisfy C1−C6.

In the following, we consider the matrix representation of permutations. A permutation matrix over $\left(C\right)$ is an *r*×*r* matrix such that $\left(\sigma_{c_{i}c_{j}}=1\right)$ if *σ*(*c*_*i*_)=*c*_*j*_, and 0 otherwise. We consider it equivalent to discuss a permutation or its corresponding matrix. Therefore, concatenation “∘” is equivalent to the matrix multiplication “·”. We use *σ*to denote a permutation or a permutation matrix, depending on the context.

**Example 1.***In genetics, the most commonly used character state set is*$\left(C_{\text{DNA}}=\{A,G,C,T\}\right)$. *There are two different Abelian groups for four states, namely the Klein-Four-group*$\left(Z_{2}\times Z_{2}\right)$*and the cyclic group*$\left(Z_{4}\right)$. *The Klein-Four-group is constructed from the cyclic group*$\left(Z_{2}\right)$*over two elements, the identityτ*_0_*and the flipτ*_1_. *These take the matrix form*

$$\tau_{0}=\left(\begin{array}{cc} 1 & 0\\ 0 & 1 \end{array}\right),\;\tau_{1}=\left(\begin{array}{cc} 0 & 1\\ 1 & 0 \end{array}\right).$$

*The Klein-Four-group consists of the four Kronecker products of these two matrices, i.e.s*_0_ = *τ*_0_ ⊗ *τ*_0_, *s*_1_ = *τ*_1_ ⊗ *τ*_0_, *s*_2_ = *τ*_0_ ⊗ *τ*_1_, and *s*_3_ = *τ*_1_ ⊗ *τ*_1_. *The Kronecker products here yield 4×4 matrices, e.g.,*

$$s_{1}=\tau_{1}⊗\tau_{0}=\left(\begin{array}{cc} 0 & \tau_{0}\\ \tau_{0} & 0 \end{array}\right)=\begin{array}{c} A\\ C\\ G\\ T \end{array}\;\overset{\begin{array}{cccc} A & C & G & T \end{array}}{\left(\begin{array}{cccc} 0 & 0 & 1 & 0\\ 0 & 0 & 0 & 1\\ 1 & 0 & 0 & 0\\ 0 & 1 & 0 & 0 \end{array}\right)}.$$

*The setΣ*_K3ST_:={*s*_0_*s*_1_*s*_2_*s*_3_} *coincides with the substitution matrices under the Kimura 3ST model*[6]. *In particular,s*_1_*describes transitions within purines* (*AG*) *and pyrimidines* (*CT*), *s*_2_*represents transversions within pairs* (*AC*) *and* (*GT*), *ands*_3_*represents the remaining set of transversions within pairs* (*AT*) *and* (*CG*).

*The second Abelian group over four states, the cyclic group*$\left(Z_{4}\right)$, *is formed by selecting a 4-cycle, e.g.,A*→*G*→*T*→*C*→*Aand concatenating this cycle with itself. The resulting set of permutations*$\left(\Sigma_{Z_{4}}\right)$*contains the following elements:*

$$\begin{array}{l} {s^{\prime }}_{1}=\begin{array}{c} A\\ C\\ G\\ T \end{array}\;\overset{\begin{array}{cccc} A & C & G & T \end{array}}{\left(\begin{array}{cccc} 0 & 0 & 1 & 0\\ 1 & 0 & 0 & 0\\ 0 & 0 & 0 & 1\\ 0 & 1 & 0 & 0 \end{array}\right)},\\ {s^{\prime }}_{2}={s^{\prime }}_{1}^{2}={s^{\prime }}_{1}\cdot {s^{\prime }}_{1},\;{s^{\prime }}_{3}={s^{\prime }}_{1}^{3},\;{s^{\prime }}_{0}={s^{\prime }}_{1}^{4}. \end{array}$$

*Note that there are actually six different four-cycles for*$\left(C_{\mathrm{DNA}}\right)$. *These result in three distinguishable Abelian groups. Bryant*[14]*generates his cyclic group with the four-cycleA*→*C*→*G*→*T*→*A*, *and shows that the resulting setΣ*_K2ST_*underlies the Kimura 2ST model*[15], *where*$\left({s^{\prime }}_{\;\;2}\right)$*corresponds to the transition within purines and pyrimidines, and*$\left({s^{\prime }}_{1}\right)$*and*$\left({s^{\prime }}_{3}\right)$*are the (not further distinguished) transversions.*

The next step in constructing the OSM matrix is to construct a set $\left(\Sigma^{T}\right)$ of operations over $\left(C^{n}\right)$ governed by $\left(T\right)$, and based on the permutation set *Σ*. To this end, we first define *Σ*^*n*^ as a set of operations which work elementwise, i.e. for $\left(f=(f_{1},\ldots ,f_{n})\in C^{n}\right)$ and *σ*∈*Σ*^*n*^ we have

$$\sigma (f):=(\sigma_{1}(f_{1}),\ldots ,\sigma_{n}(f_{n})),\;\sigma_{i}\in \mathrm{\Sigma .}$$

This can also be described by the Kronecker product, i.e. equally

(1) $$\sigma (f)=\sigma_{1}⊗\cdots ⊗\sigma_{n}(f).$$

This means that there are *r*^*n*^different operators in *Σ*^*n*^=*Σ*⊗⋯⊗*Σ*.

**Remark 1.***Therefore, for any pair of characters*$\left(f,g\in C^{n}\right)$*we can find an operationσ* ∈ *Σ*^*n*^*such thatσ* (*f*) = *g*.

Another noteworthy consequence of using the Kronecker product is that the elements of *Σ*^*n*^ are permutations over $\left(C^{n}\right)$[16,17], and in fact *Σ*^*n*^ satisfies our Conditions C1−C7, i.e. *Σ*^*n*^ is an Abelian group over $\left(C^{n}\right)$.

In the OSM framework we assume that the permutations acting on a character $\left(f\in C^{n}\right)$ are derived from the underlying rooted tree $\left(T\right)$. If permutation *σ*_*i*_∈*Σ* acts on the pendant edge leading to taxon *j*∈*X*, then the associated permutation matrix *σ*^*j*,*i*^acting on $\left(C^{n}\right)$ has the form

$$\sigma^{j,i}:=\overset{j-1}{\underset{l=1}{⊗}}\sigma_{0}⊗\sigma_{i}⊗\overset{n}{\underset{l=j+1}{⊗}}\sigma_{0}.$$

If a permutation acts on an interior edge *e*, then it simultaneously acts on the states of all descendant taxa of *e*, i.e. all those taxa whose path to the root passes *e*. E.g., assume Taxa 1 and 2 form a cherry, i.e. their most recent common ancestor, 12, has no other descendants, and permutation *σ*_*i*_∈*Σ*, *i*=1,…,*r*−1 is acting on the edge leading to this ancestor. Then, we get the permutation

(2) $$\sigma^{12,i}:=\sigma_{i}⊗\sigma_{i}⊗\sigma_{0}\cdots ⊗\sigma_{0}=\sigma^{1,i}\cdot \sigma^{2,i}.$$

This shows in particular that a Kronecker product of some permutations acting on each character state is equivalent to the matrix product of the permutations acting on the entire character. The right hand side equation shows that a single permutation on an internal edge has the same effect as simultaneously applying the same permutation on the pendant edges of all descendant taxa. In other words, if de(*e*) denotes the set of descendants of edge *e*, and *σ*_*i*_∈*Σ*, then

(3) $$\sigma^{e,i}=\underset{j\in \mathrm{de}(e)}{\prod }\sigma^{j,i}.$$

Note that the set *Σ*^*X*^of all permutations acting on the pendant edges is a generator of *Σ*^*n*^, i.e. the closure of *Σ*^*X*^ contains all permutations in *Σ*^*n*^. Since *Σ*^*n*^ contains a single permutation to transform character $\left(f\in C^{n}\right)$ into $\left(g\in C^{n}\right)$, and since *Σ*^*X*^ generates *Σ*^*n*^, there is a shortest chain of permutations in *Σ*^*X*^which transforms *f*into *g*. *Σ*^*X*^ is also the set of permutations implied by the star tree for *X*. In general, the set of all permutations on tree $\left(T\right)$ is

$$\Sigma^{T}=\left\{\sigma^{e,i}:\;e\in E(T),\;i\in \{0,\ldots ,r-1\}\right\},$$

where *r* is the number of states in *Σ*.

For every *X*-tree $\left(T\right)$ we have $\left(\Sigma^{T}⊇\Sigma^{X}\right)$, and therefore $\left(\Sigma^{T}\right)$ is a generator for *Σ*^*n*^, too. An illustration of such a generator set $\left(\Sigma^{T}\right)$ over the character set $\left(C^{n}\right)$ is the so-called *Cayley graph*[18], which has as vertices the characters of $\left(C^{n}\right)$, and two characters $\left(f,g\in C^{n}\right)$ are connected if there is a permutation $\left(\sigma \in \Sigma^{T}\right)$ such that *σ*(*f*)=*g*. In [5] Cayley graphs have been presented as alternative illustrations of the tree $\left(T\right)$ over a binary state set $\left(C=\{0,1\}\right)$.

**Figure 1:**
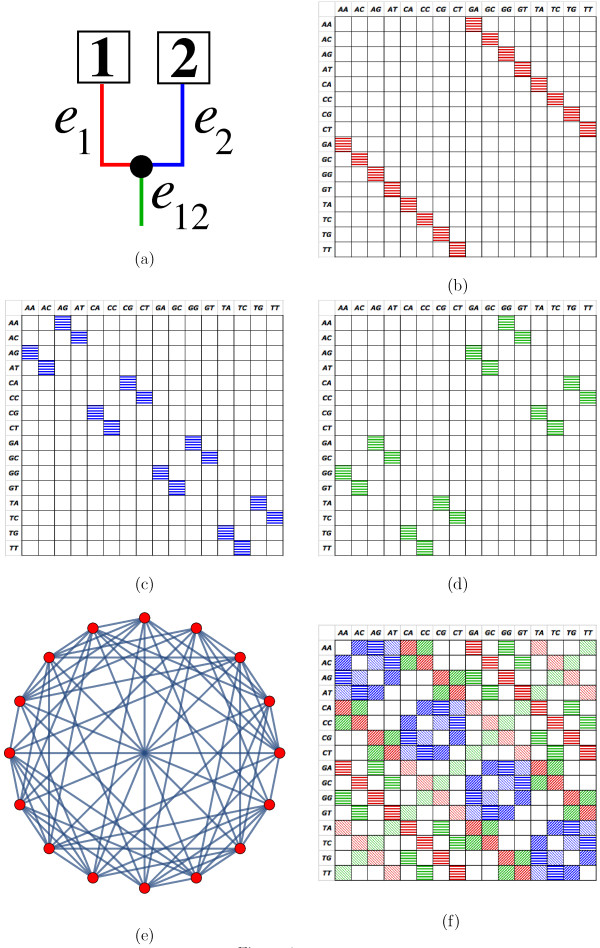
**Construction of the OSM matrix.** (**a**) A rooted tree with taxa 1 and 2. (**b**) A transition *s*_1_on the left branch *e*_1_(the red branch) changes a character into exactly one new character as depicted by the red horizontal stripe cells of the permutation matrix $\left(\sigma^{e_{1},s_{1}}\right)$. The matrix has 16 rows and 16 columns representing the possible characters for the alignment of two nucleotide sequences. The permutation matrices generated by *s*_1_for the right branch *e*_2_(blue) and for the branch leading to the “root” *e*_12_(green) are displayed in (**c**) and (**d**), respectively. The corresponding Cayley graph for the tree is illustrated in (**e**). The convex sum of all the weighted (by the relative branch length and the probability of the substitution type) permutation matrices generated by all substitution types for all branches is the OSM matrix of the tree ($\left(M_{T}\right)$) as shown in (**f**). Horizontal stripe cells represent the probability of the transition *s*_1_; diagonal stripes the transversion *s*_2_; and thin reverse diagonal stripes the transversion *s*_3_. The colors of these cells indicate the relative branch lengths and follow the colors of the branches as in (**a**). Thus, these colors also depict the branch origin of the substitutions.

**Example 2.***Regard the K3ST model from Example 1 and the rooted two-taxon tree depicted in Figure*1a. *With this*$\left(\Sigma_{\text{K3ST}}^{T}\right)$*is given by the set*

$$\begin{array}{lllllllll} s^{e_{1},1} & :=s_{1}⊗s_{0},\; & s^{e_{2},1} & :=s_{0}⊗s_{1},\; & s^{e_{12},1} & :=s_{1}⊗s_{1},\; & \; & \; & \;\\ s^{e_{1},2} & :=s_{2}⊗s_{0},\; & s^{e_{2},2} & :=s_{0}⊗s_{2},\; & s^{e_{12},2} & :=s_{2}⊗s_{2},\; & \; & \; & \;\\ s^{e_{1},3} & :=s_{3}⊗s_{0},\; & s^{e_{2},3} & :=s_{0}⊗s_{3},\; & s^{e_{12},3} & :=s_{3}⊗s_{3}.\; & \; & \; & \; \end{array}$$

Each permutation which acts on the characters is thus a symmetric 16×16 permutation matrix depicting a transition (*s*^*e*,1^), transversion 1 (*s*^*e*,2^), or transversion 2 (*s*^*e*,3^) along edge $\left(e\in E(T)\right)$. Figures 1b-d display the permutation matrices for a transition on branch *e*_1_($\left(s^{e_{1},1}\right)$), *e*_2_($\left(s^{e_{2},1}\right)$) and *e*_12_($\left(s^{e_{12},1}\right)$), respectively. Figure 1e shows the Cayley graph associated with $\left(\Sigma_{\text{K3ST}}^{T}\right)$.

We are now in a position to recall the definition of the *OSM matrix*$\left(M_{T}\right)$ for a rooted binary phylogenetic tree $\left(T\right)$ as explained in [5] and [19]. For an edge $\left(e\in E(T)\right)$ we denote by *p*_*e*_the relative branch length of *e*, i.e. its actual branch length (expected number of substitutions per site) divided by the length of $\left(T\right)$ (the sum of all branch lengths). Thus, one can view *p*_*e*_as the probability that a mutation is observed at edge *e* assuming that a single mutation occurred on $\left(T\right)$. Clearly, $\left(\underset{e\in E(T)}{\sum }p_{e}=1\right)$. Further, denote by *α*_*e*,*i*_ the probability that this mutation on *e* is of type *i*∈{1,…,*r*−1} with $\left(\overset{r-1}{\underset{i\in 1}{\sum }}\alpha_{e,i}=1\right)$ for all $\left(e\in E(T)\right)$. Then the OSM matrix is the convex sum of the elements in $\left(\Sigma^{T}\right)$, where each permutation *σ*^*e*,*i*^ is multiplied by *α*_*e*,*i*_*p*_*e*_, the probability of hitting the edge *e* with permutation *σ*_*i*_∈*Σ*. Thus, we obtain:

(4) $$M_{T}=\underset{e\in E(T)}{\sum }\overset{r-1}{\underset{i=1}{\sum }}\alpha_{e,i}p_{e}\sigma^{e,i}.$$

$\left(M_{T}\right)$ can be regarded as the weighted exchangeability matrix for all characters under the K3ST model assuming that a single substitution occurs on the tree $\left(T\right)$. Figure 1f depicts the OSM matrix for the tree in Figure 1a. Here, colors indicate relative branch lengths *p*_*e*_, and patterns denote permutation types *α*_*i*_. E.g., a blue square with horizontal lines indicates the product $\left(p_{e_{2}}\alpha_{e_{2},1}\right)$, i.e. the probability of observing a transition *s*_1_on edge *e*_2_.

### The transformation problem

With the construction of $\left(\Sigma^{T}\right)$ we have generated the tools needed to formally describe the computations in Step 4 of the Misfits algorithm [4]. Given a rooted tree $\left(T\right)$ and two characters *f*and *f*^*d*^ in $\left(C^{n}\right)$, we want to compute the minimal number of substitutions required on the tree to convert *f* into *f*^*d*^. [4] presented an efficient procedure to compute this minimal number of substitutions.

### Algorithm 1

**INPUT:** rooted binary phylogenetic tree $\left(T\right)$ on leaf set *X*, characters *f*and *f*^*d*^on *X*, Abelian group *Σ*.

**STEP 1:** Using Remark 1, find the substitution type *σ*_*i*_which translates *f*_*j*_into $\left(f_{j}^{d}\right)$ for all positions *j*=1,…,|*X*|. Let *σ*∈*Σ*^*n*^be the resulting operation, i.e. *σ*(*f*)=*f*^*d*^.

**STEP 2:** Let *c*:=*c*_1_…*c*_1_be a constant character on *X* with $\left(c_{1}\in C\right)$. Let *h*:=*σ*(*c*).

**STEP 3:** Calculate $\left(m:=l_{T}(h)\right)$.

**OUTPUT:***m*.

We prove the correctness of our algorithm. In our framework, *m* corresponds to the minimum number of permutations *σ*_1_,…,*σ*_*m*_∈*Σ* such that *σ*_1_⊗⋯⊗*σ*_*m*_(*f*)=*f*^*d*^. In this form, *m* has multiple equivalent interpretations. It is the length of the shortest path between *f* and *f*^*d*^ in the Cayley graph for $\left(\Sigma^{T}\right)$, where this path corresponds to *σ*_1_⊗⋯⊗*σ*_*m*_. Further, *m* corresponds to the minimum power (*k*) of $\left(M_{T}\right)$ such that $\left(M_{T}^{j}(f,f^{d})=0\right)$ for *j*<*k* and $\left(M_{T}^{k}(f,f^{d})>0\right)$, because a positive entry in $\left(M_{T}^{k}\right)$ means that there is a concatenation of *k* permutations connecting the associated characters.

**Figure 2:**
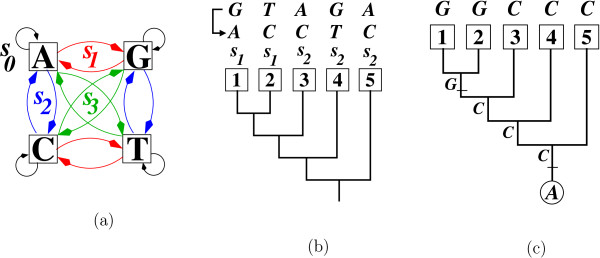
**Computing the minimal number of substitutions to translate a character into another one.** (**a**) depicts the Klein-four group *Σ*_K3ST_, which consists of the identity *s*_0_and the three substitution types *s*_1_,*s*_2_,*s*_3_from the K3ST model. (**b**) In order to convert the character *GTAGA* into *ACCTC* under *Σ*_K3ST_, we need to introduce the operation *s*:=(*s*_1_,*s*_1_,*s*_2_,*s*_2_,*s*_2_). As the operation *s* also translates the constant character *AAAAA* to *GGCCC*, converting *GTAGA* into *ACCTC* is equivalent to evolving the character state *A* at the root along the tree to obtain the character *GGCCC* at the leaves. The Fitch algorithm applied to the latter produces a unique most parsimonious solution of two substitutions as depicted by (**c**).

**Example 3.***Figure*2*demonstrates how Algorithm 4 works under the K3ST model, i.e. when the group isΣ* = *Σ*_*K3ST*_ (*Figure*2a). *Consider the rooted five-taxon tree in Figure*2b*and the character GTAGA at the leaves. Assume that the character GTAGA is to be converted into character ACCTC. By comparing the two characters position-wise, we need a substitutions*_1_*on the external branch leading to taxon 1 to convert G into A at the first position. Similarly, we need a substitutions*_1_*on the external branch leading to taxon 2, and a substitutions*_2_*on every external branch leading to taxa 3, 4, and 5. Thus, the operations*:=(*s*_1_*s*_1_*s*_2_*s*_2_*s*_2_) *transfers the character GTAGA into the character ACCTC. As the operation s also translates the constant character AAAAA into GGCCC, converting GTAGA into ACCTC is equivalent to evolving the character state A at the root along the tree to obtain the character GGCCC at the leaves. The Fitch algorithm*[7]*applied to the character GGCCC with the constraint that the character state at the root is A produces a unique most parsimonious solution of two substitutions as depicted by Figure*2c. 

## Results

### The impact of parsimony on the estimation of substitutions

In this section, we provide some mathematical insights into the role of maximum parsimony in the estimation of the number of substitutions needed to convert a character into another one as explained above. In particular, we deliver a proof for Algorithm 4.

**Theorem 1.** Let $\left(T\right)$*be a rooted binary phylogenetic tree on taxon set X and let f be a character that evolved on*$\left(T\right)$*due to some evolutionary model and letf*^*d*^*be another character on X. Then, the minimum number of substitutions to be put on*$\left(T\right)$*which change the evolution of f in such a way thatf*^*d*^*is generated can be calculated with Algorithm 4.*

#### Proof

Let *f*, *f*^*d*^, *X*, $\left(T\right)$ and *Σ*be as required for the input of Algorithm 4. Then, as defined in the algorithm, we have $\left(\hat{\sigma }(f)=({\hat{\sigma }}_{1}(f_{1}),{\hat{\sigma }}_{2}(f_{2}),\ldots ,{\hat{\sigma }}_{n}(f_{n}))=f^{d}\right)$, where $\left({\hat{\sigma }}_{j}\in \Sigma \right)$ refers to the substitution type needed to translate *f*_*j*_into $\left(f_{j}^{d}\right)$.

Considering the underlying tree $\left(T\right)$, we may assume $\left({\hat{\sigma }}_{1},\ldots ,{\hat{\sigma }}_{n}\right)$ act on the pending branches leading to taxa 1,…,*n*, respectively.

Now we show that it is equivalent to consider $\left(\hat{\sigma }(c)\right)$, where *c* is a constant character, instead of $\left(\hat{\sigma }(f)\right)$. Let $\left(\mu \in \Sigma^{T}\right)$ be a transformation with *μ*(*f*)=*f*^*d*^. Then,

(5) $${\hat{\sigma }}^{-1}\circ \mu (f)={\hat{\sigma }}^{-1}(f^{d})=\mathrm{f.}$$

Next, let $\left(\tilde{\sigma }\in \Sigma^{T}\right)$ be such that $\left(\tilde{\sigma }(c)=f\right)$. Then, using (5), we have

$${\hat{\sigma }}^{-1}\circ \mu \circ \tilde{\sigma }(c)={\hat{\sigma }}^{-1}\circ \mu (f)=f=\tilde{\sigma }(c).$$

On the other hand, we can use the commutativity of the underlying Abelian group to derive

$${\hat{\sigma }}^{-1}\circ \mu \circ \tilde{\sigma }(c)=\tilde{\sigma }\circ {\hat{\sigma }}^{-1}\circ \mu (c).$$

So altogether we have

$${\hat{\sigma }}^{-1}\circ \mu \circ \tilde{\sigma }(c)=\tilde{\sigma }\circ {\hat{\sigma }}^{-1}\circ \mu (c)=\tilde{\sigma }(c)$$

and therefore $\left({\hat{\sigma }}^{-1}\circ \mu (c)=c\right)$ and thus $\left(\mu (c)=\hat{\sigma }(c)\right)$. As *μ*was arbitrarily chosen, this implies that any transformation which maps *f* to $\left(\hat{\sigma }(f)=f^{d}\right)$ also maps *c* to $\left(\hat{\sigma }(c)\right)$. Therefore, we have

$$\{\rho \in \Sigma^{T}:\rho (f)=f^{d}\}=\{\rho \in \Sigma^{T}:\rho (c)=\hat{\sigma }(c)\}.$$

The minimum number of substitutions to change *f*from *f*^*d*^ on $\left(T\right)$ is just an element of the first set consisting of the fewest number of compositions. As the two sets are equal, we can investigate the second set rather than the first. So we need an element of the second set which consists of as few as possible compositions. Assuming that *σ*=*σ*_1_⊗⋯⊗*σ*_*n*_, we can assign *σ*_1_,…,*σ*_*n*_ to the pending branches of $\left(T\right)$ and treat them like character states to which we then apply the Fitch algorithm. This completes the proof. □

Informally speaking, the idea is as follows: As there is exactly one path from the root *ρ* to any taxon *x*∈*X*, we wish to determine whether we can ‘pull up’ some of the operations along this path in order to affect more than one taxon and still give the same result. This idea has been described above (Equations (2) and (3)), and it coincides precisely with the idea of the parsimony principle.

However, in order to avoid confusion regarding the operation *σ* as a character on which to apply parsimony, Algorithm 4 instead acts on the constant character. Clearly, in order to evolve the constant character *c*:=*c*_1_⋯*c*_1_ on a tree with root state *c*_1_, the corresponding operation would be $\left(\tilde{\sigma }:=\sigma_{0}⊗\cdots ⊗\sigma_{0}\right)$. Note that *σ*(*c*)=*h* and *σ*(*f*)=*f*^*d*^, and that two character states in *h*are identical if and only if the corresponding substitutions in *σ* are identical, too. Therefore, it is possible to let MP act on *h*rather than directly on *σ*.

By the definition of maximum parsimony, when applied to *h* on tree $\left(T\right)$ with given root state *c*_1_, it calculates the minimum number *m* of substitutions to explain *h*on $\left(T\right)$. This number *m* is therefore precisely the number of substitutions needed to generate *h* on $\left(T\right)$ rather than *c*. As *σ*(*f*)=*f*^*d*^, *m* also is the number of substitutions needed to generate *f*^*d*^from *f* on $\left(T\right)$.

### The impact of different groups

For any alphabet $\left(C\right)$, there might be more than one Abelian group. Different groups might result in different numbers of substitutions required to translate a character into another character. We illustrate this observation using the following example.

**Figure 3:**
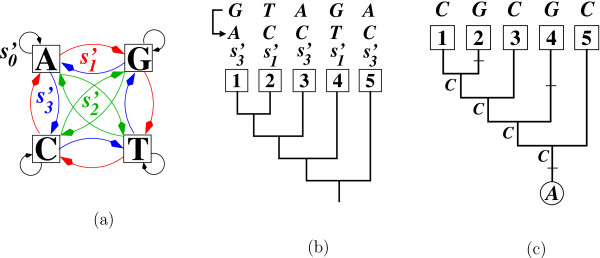
**Converting one character into another character using the cyclic group.** (**a**) depicts the cyclic group *Σ*_*c*_, which consists of the identity $\left(s_{0}^{\prime }\equiv s_{0}\right)$ and the three substitution types $\left(s_{1}^{\prime },s_{2}^{\prime },s_{3}^{\prime }\right)$ for nucleotide character states. (**b**) In order to convert the character *GTAGA* into *ACCTC* using this group, we need to introduce the operation $\left(s^{\prime }:=(s_{3}^{\prime },s_{1}^{\prime },s_{3}^{\prime },s_{1}^{\prime },s_{3}^{\prime })\right)$. As the operation *s*^*′*^also transforms the constant character *AAAAA* to *CGCGC*, converting *GTAGA* into *ACCTC* is equivalent to evolving the character state *A* at the root along the tree such that the character *CGCGC* is attained at the leaves. The Fitch algorithm applied to the latter produces a unique most parsimonious solution of three substitutions as depicted by (**c**).

**Example 4.***Recall the starting point of Example 3, i.e. regard the five-taxon tree T from Figure*3b, *and the characters f = GTAGA and f*^*d*^ = *ACCTC. Now, instead of usingΣ*_K3ST_*we use the permutations from the cyclic group*$\left(\Sigma_{Z_{4}}\right)$. *In this setting, we need a substitution*$\left({s^{\prime }}_{3}\right)$*(blue in Figure*3a*) on the external edge leading to taxon 1 to convert G into A at the first position, and so on. Thus, we get the operation*$\left({s^{\prime }}_{2}:=({s^{\prime }}_{3},{s^{\prime }}_{1},s_{3}^{\prime },s_{1}^{\prime },{s^{\prime }}_{3})\right)$*such thats*^*′*^ (*f*) = *f*^*d*^. *We immediately see, thats*^*′*^*transforms the constant character c = AAAAA into h = CGCGC. The Fitch algorithm applied to the character CGCGC with the constraint that the character state at the root is A produces a unique most parsimonious solution of three substitutions as depicted by Figure*3c. *Thus, under theΣ*_*c*_*group we need one substitution more than under theΣ*_K3ST_*group (cf. Example 3).*

Note that variation of the minimum number of substitutions needed to translate a character into another one between different groups is not surprising: As different substitution types are needed to translate one pattern into the other one, depending solely on the underlying group, one group might need the same substitution type for some neighboring branches in the tree and another group different ones. Informally speaking, this would imply that in the first case, the substitution could be “pulled up” by the Fitch algorithm to happen on an ancestral branch, whereas in the second case this would not be possible.

### The link between substitution models and permutation matrices

In Examples 1 and 2 we have shown that the K3ST substitution model can be included into our framework. The connection between the Klein-Four-group and the K3ST model (as well as the one between the $\left(Z_{2}\right)$ group and symmetric 2-state model) were described in-depth in [9]. This section aims at discussing alternative models and how to identify their use (or lack thereof) for our approach.

Most substitution models assume the independence of the different branches of a tree to compute the joint probability of the characters in $\left(C^{n}\right)$. Therefore, they use the probabilities for substitutions among the character states in $\left(C\right)$ along the edges of the tree $\left(T\right)$. We now establish a probabilistic link between $\left(\Sigma^{T}\right)$ and $\left(C^{n}\right)$. This link is provided by Birkhoff’s theorem:

**Theorem 2 (Birkhoff’s theorem, e.g.,**[20], **Theorem 8.7.1).***A matrix M is doubly stochastic, i.e., each column and each row of M sum to 1, if and only if for some N < ∞ there are permutation matricesσ*_1_, …, *σ*_*N*_*and positive scalarsα*_1_, …, *α*_*N*_ ∈ *[0,1] such thatα*_1_ + ⋯ + *α*_*n*_* = 1 and M = α*_1_*σ*_1_ + ⋯ + *α*_*N*_*σ*_*N*_.

Therefore, the weighted sum of the permutation matrices in $\left(\Sigma^{T}\right)$ yields a doubly stochastic matrix $\left(M_{T}\right)$ as introduced above. $\left(M_{T}\right)$ also describes a random walk on $\left(C^{n}\right)$ governed by $\left(T\right)$ where the single step in $\left(C^{n}\right)$ is illustrated by the associated Cayley graph. Its stationary distribution is uniform, i.e. when we throw sufficiently many mutations on $\left(T\right)$ then we expect to see each character with probability 1/*r*^*n*^.

Another, even more useful consequence of Birkhoff’s theorem is the fact that it tells us which substitution models are suited for the OSM approach. If the transition matrix associated with the substitution model is doubly stochastic, then we find a set of permutations which give rise to the model.

Let us see how this influences the symmetric form of the general time reversible model (sGTR) with uniform stationary distribution. It has the transition probability matrix

$$P_{\mathrm{sGTR}}=\begin{array}{c} A\\ C\\ G\\ T \end{array}\;\overset{A}{(\begin{array}{c} 1-a-b-c\\ a\\ b\\ c \end{array}}\;\overset{C}{\begin{array}{c} a\\ 1-a-d-e\\ d\\ e \end{array}}\;\overset{G}{\begin{array}{c} b\\ d\\ 1-b-d-f\\ f \end{array}}\;\overset{T}{\begin{array}{c} c\\ e\\ f\\ 1-c-e-f \end{array})}.$$

Assigning permutation matrices to the respective parameters yields the set *Σ*_sGTR_ with elements *s*_0_ (identity) and

$$\begin{array}{llllll} s_{a} & =\left(\begin{array}{cccc} 0 & 1\; & 0 & 0\\ 1 & 0\; & 0 & 0\\ 0 & 0\; & 1 & 0\\ 0 & 0\; & 0 & 1 \end{array}\right),\; & s_{b} & =\left(\begin{array}{cccc} 0 & 0\; & 1 & 0\\ 0 & 1\; & 0 & 0\\ 1 & 0\; & 0 & 0\\ 0 & 0\; & 0 & 1 \end{array}\right)\; & \; & \;\\ s_{c} & =\left(\begin{array}{cccc} 0 & 0\; & 0 & 1\\ 0 & 1\; & 0 & 0\\ 0 & 0\; & 1 & 0\\ 1 & 0\; & 0 & 0 \end{array}\right),\; & s_{d} & =\left(\begin{array}{cccc} 1 & 0\; & 0 & 0\\ 0 & 0\; & 1 & 0\\ 0 & 1\; & 0 & 0\\ 0 & 0\; & 0 & 1 \end{array}\right),\; & \; & \;\\ s_{e} & =\left(\begin{array}{cccc} 1 & 0\; & 0 & 0\\ 0 & 0\; & 0 & 1\\ 0 & 0\; & 1 & 0\\ 0 & 1\; & 0 & 0 \end{array}\right),\; & s_{f} & =\left(\begin{array}{cccc} 1 & 0\; & 0 & 0\\ 0 & 1\; & 0 & 0\\ 0 & 0\; & 0 & 1\\ 0 & 0\; & 1 & 0 \end{array}\right).\; & \; & \; \end{array}$$

The weighted sum of the non-identity elements yields

$$\begin{array}{lll} as_{a} & +bs_{b}+cs_{c}+ds_{d}+es_{e}+fs_{f}\; & \;\\ & =\left(\begin{array}{cccc} d+e+f & a & b & c\\ a & b+c+f & d & e\\ b & d & a+c+e & f\\ c & e & f & a+b+d \end{array}\right),\; & \; \end{array}$$

which is equal to *P*_sGTR_if *a* + *b* + *c* + *d* + *e* + *f*=1. Thus, the set *Σ*_sGTR_is to sGTR what *Σ*_K3ST_ is to K3ST. However, *Σ*_sGTR_ does not satisfy condition C5, because *s*_*a*_,⋯, *s*_*f*_ are not fixed point free. This can be seen as the main diagonal of *s*_*a*_,⋯, *s*_*f*_does not only contain zeros. It is also not commutative (condition C4) as e.g. *s*_*a*_·*s*_*c*_≠*s*_*c*_·*s*_*a*_. And it is not closed under matrix multiplication (condition C3), which means that a concatenation of permutations in *Σ*_sGTR_ might lead to a new permutation not in *Σ*_sGTR_, e.g., *s*_*a*_·*s*_*f*_∉*Σ*_sGTR_. Other complex models like Tamura-Nei [21] do not even permit the decomposition of its transition matrix into the convex sum of permutation matrices. All of this shows why the overall applicability of complex models to the OSM approach is rather limited.

There are other approaches to describe phylogenetic models based on the group structure of their substitution matrices. In particular, Sumner et al. [22] use Lie algebra to construct OSM type matrices for the general Markov model, and discuss shortcomings of the group structure for the general GTR model [23].

### Application to other biologically interesting sets

As stated above, OSM-type models require an underlying Abelian group. Thus, the OSM setting is applicable not only to binary data or four-state (DNA or RNA) data, but also to alphabets of 16 (doublets), 64 (codons), and 20 characters (amino acids) respectively. We compare such extensions to existing biologically motivated binning approaches and discuss their relevance.

As we have shown in the previous sections, the symmetric form of the Klein-Four-Group $\left(Z_{2}\times Z_{2}\right)$ is mathematically beautiful, computationally convenient and biologically relevant. Similar statements can be made about all powers of $\left(Z^{2}\right)$, including the biologically relevant alphabets of 16 (doublets) and 64 (codons) letters.

There are four Abelian groups for twenty-state alphabets, namely $\left(Z_{2}\times Z_{2}\times Z_{5},\;Z_{4}\times Z_{5},\;Z_{2}\times Z_{10}\right)$, and the cyclic group $\left(Z_{20}\right)$ (see e.g., [24] for a complete list of all groups with up to 35 elements). Their construction is analogous to the construction of the Klein-Four-group in Example 1. For example, the elements of $\left(Z_{4}\times Z_{5}\right)$ are Kronecker products of one of the four permutations in the cyclic group $\left(Z_{4}\right)$ with one of the five permutations of the cyclic group $\left(Z_{5}\right)$.

**Figure 4:**
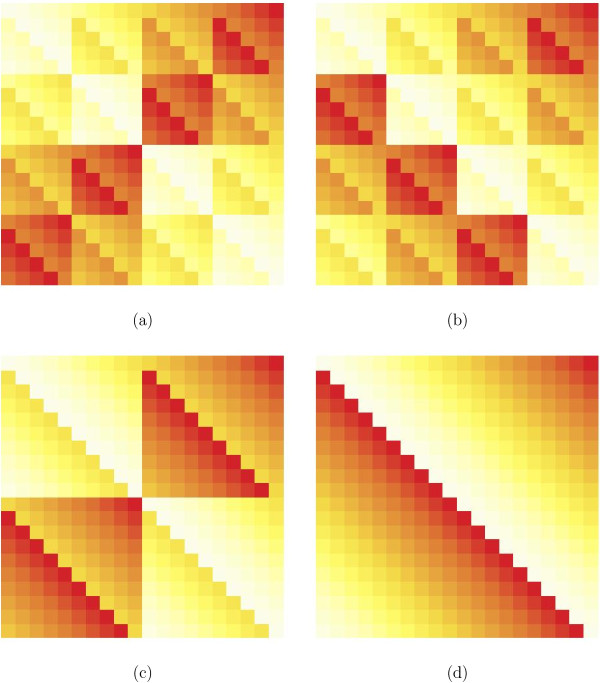
**Matrices illustrate the four Abelian groups for a twenty-state alphabet.** (**a**) the $\left(Z_{2}\times Z_{2}\times Z_{5}\right)$ group, (**b**) $\left(Z_{4}\times Z_{5}\right)$, (**c**) $\left(Z_{2}\times Z_{10}\right)$, and (**d**) $\left(Z_{20}\right)$. Each matrix visualizes the cosets of the subgroups of the depicted group and suggests an associated grouping of the 20 states.

Figure 4 shows a heat-map type visualization of an OSM-type matrix on a single-leaf tree where the coloring of the cells corresponds to the weights given to the 20 permutations in the respective groups. We see that the coloring pattern nicely reflects the four cosets of the subgroup $\left(Z_{5}\right)$ in $\left(Z_{2}\times Z_{2}\times Z_{5}\right)$. This can also be interpreted as a binning of the 20 states in the underlying alphabet into four sets of five elements each. If the weighting corresponds to a convex combination of operations, then the visualized matrix is doubly stochastic.

Binnings are also done for amino acids, using either biochemical properties or evolutionary divergence. An example of a biochemical binning is the hydrophobic index, where the 20 amino acids are binned into four groups, very hydrophobic, hydrophobic, neutral, and hydrophilic. Unfortunately, this binning does not correspond to any of the proposed Abelian groups. Moreover, it is difficult to derive transitions between these groups just from the biochemical properties.

Transition matrices for evolutionary models for amino acid substitutions are usually generated by counting mutation types in the alignments (see, e.g., [25] for an overview). From these, optimal groupings can be obtained using clustering approaches [26]. The existence of estimates for the transition probability between all amino acids provides the possibility to get further information about between-group operations. These groupings could be forced to fit Abelian groups. However, as indicated in [26] a grouping into four groups of five amino acids each is rarely optimal.

## Conclusions

In this paper, we provide the necessary mathematical background for the OSM setting which was introduced and used previously [4,19], but had not been analyzed mathematically for more than two character states. Moreover, the present paper also delivers new insight concerning the requirements for the OSM model to work: In fact, we were able to show that mathematically, it is sufficient to have an underlying Abelian group – which shows a generalization of the OSM concept that was believed to be impossible previously [4]. Therefore, we show that OSM is applicable to any number of states.

However, note that the original intuition of the authors in [4] was biologically motivated: The authors supposed that the group not only has to be Abelian, but also symmetric in the sense that each operation can be undone by being applied a second time. Thinking about DNA, for instance, this works: For example, the transition from A to G can be reverted by another substitution of the same type, namely a transition from G to A. This symmetry condition is fulfilled by the Klein-Four-group, but not by the cyclic group on four states.

While the OSM approach can be extended to any number of states, its biological relevance becomes somewhat obscure when there is no corresponding group which is a power of $\left(Z^{2}\right)$. In particular, there are four distinct Abelian groups for 20 states, but none fits a biologically meaningful binning of the 20 amino acids.

## Competing interests

The authors declare no competing interests.

## Authors’ contributions

All authors contributed equally. All authors read and approved the final manuscript.
